# *Stenotrophomonas maltophilia*: The Landscape in Critically Ill Patients and Optimising Management Approaches

**DOI:** 10.3390/antibiotics13070577

**Published:** 2024-06-22

**Authors:** Nieves Carbonell, María Rosa Oltra, María Ángeles Clari

**Affiliations:** 1 Medical Intensive Care Unit, Clinic University Hospital, INCLIVA Biomedical Research Institute, 46010 Valencia, Spain; 2 Infectious Disease Unit, Internal Medicine Department, Clinic University Hospital, INCLIVA Biomedical Research Institute, 46010 Valencia, Spain; mrosaoltra@gmail.com; 3 Microbiology Service, Clinic University Hospital, INCLIVA Biomedical Research Institute, 46010 Valencia, Spain; maclapons@hotmail.com

**Keywords:** ICU, critically ill, sepsis, antimicrobial, multidrug-resistant bacteria, sepsis, antimicrobial stewardship, *Stenotrophomonas maltophilia*

## Abstract

The aim of this review is to synthesise the key aspects of the epidemiology, current microbiological diagnostic challenges, antibiotic resistance rates, optimal antimicrobial management, and most effective prevention strategies for *Stenotrophomonas maltophilia* (SM) in the intensive care unit (ICU) population. In recent years, resistance surveillance data indicate that SM accounts for less than 3% of all healthcare-associated infection strains, a percentage that doubles in the case of ventilator-associated pneumonia (VAP). Interestingly, SM ranks as the third most isolated non-glucose fermenter Gram-negative bacilli (NFGNB). Although this NFGNB genus has usually been considered a bystander and colonising strain, recently published data warn about its potential role as a causative pathogen of severe infections, particularly pneumonia and bloodstream infections (BSI), not only for the classical immunocompromised susceptible host patients but also for critically ill ones even without overt immunosuppression. Indeed, it has been associated with crude 28-day mortality as high as 54.8%, despite initial response following targeted therapy. Additionally, alongside its intrinsic resistance to a wide range of common antimicrobials, various worldwide and local surveillance studies raise concerns about an increase in ICU settings regarding resistance to first-line drugs such as cotrimoxazole or tigecycline. This scenario alerts ICU physicians to the need to reconsider the best stewardship approach when SM is isolated in obtained samples from critically ill patients. Despite the coverage of this multidrug-resistant bacterium (MDRB) provided by some traditional and a non-negligible number of current pipeline antimicrobials, an ecological and cost-effective strategy is needed in the present era.

## 1. Introduction

The genus *Stenotrophomonas* comprises at least eight species, of which *Stenotrophomonas maltophilia* (SM) is the most predominant and the only one known to cause human disease [1]. SM is an environmental non-glucose fermenter Gram-negative bacillus (NFGNB) isolated from aqueous-associated sources both inside and outside the hospital setting [2]. It is ubiquitous and often encountered as a coloniser of plants and medical devices owing to its site adhesion and biofilm formation abilities [3]. Not typically found in the common gut flora, it can colonise the oral cavity and the respiratory tract epithelial cells of cystic fibrosis (CF) and hospitalised patients, causing chronic infection of the airways in this population that contributes to inflammation, lung damage, and premature mortality. 

Interestingly, although understudied, SM ranks as the third most isolated unusual NFGNB (after *Pseudomonas aeruginosa* and *Acinetobacter baumannii*) in various reports [4,5]. Most SM isolates are hospital-acquired, with a median delay of 8–14 days of hospital stay, but community-acquired isolates have also been described [4,6]. 

To date, distinguishing between SM colonisation and true infection represents a significant challenge, especially when more than one pathogen is isolated. This yet unresolved issue needs to be elucidated because although other host and severity clinical factors enable physicians to tailor management decision-making, finding a balance between antimicrobial overuse and the risk of worse prognosis due to absent or delayed treatment remains complicated [7]. SM infection rates have been confirmed to range from 13 to 60% according to definitions for surveillance of healthcare-associated infection established by the National Healthcare Safety Network managed by the Centers for Disease Control and Prevention (CDC/NHSN) [8,9]. Moreover, in a prospective multicentre cohort (33% ICU patients), Fihman V et al. observed that SM caused infection more often than colonisation [RR 1.74 (95% CI 1.15–2.63), *p* = 0.01] [4]. In a previous review of 13 cohort studies, a considerable mortality rate (up to 37.5%) was attributed to SM infection. These authors warned clinicians against underestimating its clinical significance, even redefining the term “SM colonisation” [10].

As an unusual NFGNB, SM has often been considered less pathogenic than many other nosocomial organisms. However, this is without considering immunocompromised patients or those with chronic respiratory diseases such as CF, who represent a debilitated population at high risk of suffering from severe and mainly nosocomial infections from this opportunistic pathogen [4,11]. 

Furthermore, SM infections are notable in the ICU population. All patients admitted to ICU are at high risk of infection by MDRB, even without a known immunosuppressed status, and when infected, need timely appropriate treatment due to an increased risk for mortality. In an earlier, classic study in 1995, Laing FPY et al. found that ICU patients were more frequently infected than those in non-ICU settings [12]. Although ICU-acquired colonisation and/or infection related to SM was considered uncommon among immunocompetent ICU patients (two patients per 1000 ICU days) [6], it was nonetheless recognised as a potentially worrisome emerging ICU pathogen; indeed, an ICU-acquired infection related to SM was independently associated with ICU mortality in this study. A recently published study confirms this ominous SM infection prognosis. It has been associated with crude 28-day mortality as high as 54.8% despite initial response after targeted therapy, especially if respiratory-focused [13]. 

Several studies have investigated the risk factors for SM infection in ICU patients, some of which are related to illness severity and underlying comorbidities such as APACHE-II score > 20, older age, and chronic obstructive pulmonary disease (COPD). Other risk factors that are concerned with invasive procedures at ICU admission include indwelling intravascular catheter devices, mechanical ventilation, tracheotomy, and tracheal intubation. Finally, SM isolation within 30 days, hospitalisation within the last three months, and prolonged antibiotic treatment (specially carbapenems [CP], but also β-lactamase inhibitors, antipseudomonal cephalosporins and aminoglycosides) have been associated with SM infections; COPD and CP use have been demonstrated as independently predictive factors [4,6,7,11,14,15,16]. 

Defining the clinical risk factors for SM infection is, therefore, a challenge, but one that would be highly beneficial for adequate antimicrobial treatment in the current MDRB era. Initial CP regimens covering extended-spectrum beta-lactamase-producing *Enterobacteriaceae* (ESBL-PE) bacteria causing community and healthcare infections could lead to inappropriate SM treatment due to its intrinsic CP-resistant profile. Indeed, an association between inappropriate initial antimicrobial treatment and mortality has previously been reported in SM-infected, either critically ill or non-critical cases [4,6,10].

This current situation is a challenge for doctors, who need to raise the index of suspicion. Moreover, knowledge about local epidemiology is essential to become familiar with antibiotic resistance rates and accordingly plan an optimal antimicrobial stewardship strategy. Finally, updated infection control measures such as those included in the Spanish “Zero Resistance” program [17] should accompany these bundles to improve the prognosis of SM infections in ICU patients.

This review aims to highlight key issues over the last twenty years to help provide clinical guidance for when SM is isolated in critically ill patients.

## 2. Relevant Sections

### 2.1. Commensal vs. Pathogenic Status of SM: Relevant Aspects

#### 2.1.1. Why Should We Consider Highlighting the Importance of Colonisation by SM?

While microbial culture isolation of a putative pathogen in the laboratory is often associated with infectious syndromes such as BSIs and other sterile body site infections, the correlation is not well defined for NFGNB when the cultured specimen is from body sites with a high colonisation/contamination potential, especially when more than one pathogen is isolated [7]. 

In contrast, colonisation by an MDRB has been associated with a significantly higher frequency of subsequent infection with the same MDRB and increased mortality in the ICU population. A meta-analysis including a total of 13 studies (with 15,045 ICU patients) found the risk for subsequent ESBL-PE infection to be almost 50-fold higher in colonised than non-colonised patients. These authors concluded that ESBL-PE colonisation of the digestive tract could be a useful marker to predict ESBL-PE infection [18]. In a single-centre retrospective observational study including 3463 surveillance cultures from 250 haematological patients (11 from ICU), Torres I and colleagues documented MDRB colonisation in 33.7% of admissions, most frequently by NFGNB (55.7%). Colonisation by any MDRB was independently associated with an increased risk of MDRB-BSI (HR, 3.70; 95% CI, 1.38–9.90; *p* = 0.009) [19].

SM is rarely present in the oropharynx microbiome of healthy patients but can often be recovered from the oropharynx of hospitalised and CF patients. In the specific case of SM, infection was predicted (96% sensitivity and 93% specificity) by a relative abundance of 36% of oral *Stenotrophomonas*, as assessed via 16SrRNA gene quantification in a cohort of 90 acute myeloid leukaemia (AML) patients receiving remission induction chemotherapy [14]. An SM infection incidence of 9% was reported in this immunosuppressed non-ICU population. In a previous similar study, which analysed SM colonisation during allogeneic hematopoietic stem cell transplantation, the authors observed that SM colonised patients had a worse prognosis, with a poorer overall survival from 6 to 60 months due to higher non-relapsed mortality after allo-transplant (6 months: 15% vs. 4.8% and 60 months: 40.1% vs. 16.2%, *p* = 0.003) [20]. SM colonised in the oral pharynx tends to form bacterial biofilms in the lining of indwelling catheters, increasing the risk of pulmonary SM infection. The results obtained from immunosuppressed non-ICU patients are quite different from those described with *Enterobacterales*, non-SM NFGNB, and *Enterococcus* spp., which usually involve the importance of negative predictive value for suspected infection, frequently BSI caused by gastrointestinal translocation due to rectal colonisation [18,19].

Several classic prospective case-control studies in the ICU population contain significant findings and suggestions focused on the patient risk profile and outcomes of SM infection. Regarding the former, four studies stand out. First, research performed by the Grupo Andaluz para el Estudio de Enfermedades Infecciosas (GAEI) in six tertiary hospitals (with one-third of patients in ICU) revealed a pooled incidence of SM colonisation or infection of 5.7 cases per 10,000 admissions. About half were considered infected, with pneumonia as the most frequent type of infection in ICU patients (two-thirds VAP). The results of qualitative and quantitative analysis of variables independently associated with an increased risk of acquiring SM pointed to SM as a potential cause of nosocomial infections in patients who had previously received a prolonged course of a carbapenem, ceftazidime, or quinolone, particularly if they were under mechanical ventilation [9]. Second, a more recently published meta-analysis including 2320 ICU patients, of which 306 (13%) were SM-infected, showed a particular risk profile with highly suspected SM origin in ICU-acquired pneumonia, specifically including the above-mentioned criteria. These authors drew attention to a subset of critically ill patients with underlying comorbidities (COPD, malignancy, and high APACHE-II score) who had undergone invasive procedures related to artificial airways and were on broad-spectrum antibiotics due to a combination of host and medical factors [15]. Third, during the pandemic period, a systematic Japanese review of COVID-19-associated SM infection (most patients requiring ICU admission) reported these patients as having similar risk factors as in the above-mentioned studies of patients without COVID-19 [21]. Finally, revisiting the risk factors for the second most common SM infection, bacteraemia, a retrospective study comparing 54 SM with 167 *P. aeruginosa* and 69 *Acinetobacter* species bacteraemic patients showed SM isolation within 30 days prior to infection onset as an independent risk factor for SM bacteraemia, with earlier SM isolation in 66.7%. Nearly half of these patients were considered to have secondary bacteraemia. The thirty-day all-cause mortality of 33.3% among the SM group was significantly higher than that of the other NFGNB groups, and catheter-related infections were associated with survival [16].

Likewise, the outcomes of SM infections acquired during ICU stay have prompted clinical physicians to consider antimicrobial treatment. In a prospective observational case-control study among immunocompetent cases with ICU-acquired infection related to SM (2% of overall incidence), 80% of patients had prior colonisation related to this microorganism [6]. The study reported a significantly higher mortality rate in cases with ICU-acquired SM infection than those with ICU-acquired SM colonisation (70% vs. 25%, OR = 2.5 [1.2 to 4.9], *p* = 0.029). Furthermore, ICU-acquired SM infection was independently associated with ICU mortality. Despite evidence suggesting that clinicians should consider the importance of the predictive value of proven colonisation by SM in critically ill patients, there is no evidence supporting the benefit of antimicrobial use; instead, it likely incurs an ecological cost in this scenario. Therefore, close monitoring of these colonised patients should be considered a daily best practice.

#### 2.1.2. Why and When Should We Consider SM as a Real-Life Pathogen in the ICU? The Importance of VAP Cases in the Critically Ill

SM can cause serious infections, including bacteremia, tracheobronchitis, pneumonia, meningitis, endocarditis, endophthalmitis, skin and soft tissue, urinary tract, and oral cavity infections. Although this bacterium is normally nosocomial, community-onset infections have also been reported [3]. Worldwide, the most common clinically observed specimen is blood, with a frequency of 36.84%, followed by the eye and respiratory tract (19.29% and 15.78%, respectively) [11]. In the classic multicentre case-control study from the GAEI group, the authors underline a difference between ICU and cancer patients, with the most frequent infections being respiratory (especially VAP) in the former and primary or catheter-related bacteriemia in the latter [9]. 

From a global perspective, according to CHINET bacterial resistance surveillance data, in 2020, SM accounted for 2.98% of all strains and ranked third among NFGNB [5]. In the same way, in the recently published European surveillance, SM accounted for 0.8% of all healthcare-associated infections, rising to 1.9% in cases of pneumonia/lower respiratory infections [22]. Likewise, it is noteworthy that SM is usually linked to pulmonary infections in critically ill patients. In fact, in a 2021 CDC/NHSN updated national summary of pathogens distributions, SM appeared among the most frequently possible VAP pathogens not usually reported from other device-associated infections, accounting for 4% in hospital ICUs and ranking ninth for this type of healthcare infection. Similarly, in the Spanish national surveillance programme of ICU-acquired infection (ENVIN-HELICS registry) in 2023, SM represented 2.68% of isolated microorganisms among the main ICU-acquired infections. SM was especially important in the case of VAP (5.88%), ranking fourth after *P. aeruginosa*, *Klebsiella pneumoniae*, and *Staphylococcus aureus* [23].

Besides the above-mentioned risk factors for SM infections in critically ill patients, some evidence points to the length of ICU stay as associated with acquiring SM during hospitalisation, suggesting that empiric treatment should be considered for this microorganism in this setting. The GAEI group described a median duration of 17 days (range 0–180) before SM isolation [9]. A subsequent study reported a mean time of 14 ± 11 days between ICU admission and the first ICU-acquired SM [6]. The duration of invasive procedures (such as mechanical ventilation >14 days) also seems important, having been identified as an independent risk factor for ICU-acquired SM infection in a neurological ICU [24].

In a recently published retrospective bi-centre study including 103 ICU patients with SM infection, pneumonia (hospital-acquired pneumonia [HAP] and VAP) was the predominant clinical syndrome (72.8%), while 22% of cases were in haemato-oncology patients. In multivariable analysis, only increasing age and haemato-oncologic disease were shown to be independent risk factors for 28-day mortality SM infection, which was as high as 54.8% despite initial clinical and laboratory response after targeted therapy [13]. 

The possibility of developing fatal haemorrhagic pneumonia caused by SM has also been observed in the population with hematologic malignancies with SM bacteraemia. It is a rare presentation, with only 91 reported cases worldwide from 1990 to 2023, but with a very high associated 30-day mortality of 90.1%, even after prompt antibiotic treatment [25,26,27]. Due to its severity and rapid progression, after analysing the few survival cases, some authors emphasise the importance of early suspicion and suggest combination therapy, including TMP-SMX, for this clinical picture in this specific population, although no clinical studies are available [25,26]. The exact mechanism by which SM causes haemorrhage is unknown. Windhorst et al. propose pathogenic factors such as StmPr1, a protease with broad specificity secreted by SM, leading to tissue invasion, destruction, and haemorrhage [28]. Moreover, neutropenia (<500/mm^3^) and thrombocytopenia (<50,000/mm^3^) have been identified as risk factors for haemorrhagic pneumonia, which could partly explain the bleeding progression in patients with haematologic malignancies with SM bacteraemia [25].

### 2.2. Antibiotic Resistance Traits and Microbiological Diagnostic Challenges: Clinical Impact on the ICU Population

#### 2.2.1. Basis of SM Antibiotic Resistance 

Numerous intrinsic and acquired resistance traits make SM-caused infections notoriously difficult to treat. This bacterium is intrinsically resistant to a wide range of antibiotics, including most β-lactams, fluoroquinolones, tetracycline derivatives (except for doxycycline, minocycline, or tigecycline), chloramphenicol, all aminoglycosides (including kanamycin, tobramycin, amikacin, and neomycin) and trimethoprim [29]. SM employs a wide spectrum of antibiotic resistance mechanisms, which include overexpression/mutation of multidrug resistance efflux pumps, reduced membrane permeability, the chromosomally encoded Sm*qnr* gene (that protects both gyrase and topoisomerase IV from quinolones), and the production of beta-lactamase, carbapenemase, and aminoglycoside-modifying enzymes [11,29]. It is noteworthy that the high carbapenem resistance of SM is primarily caused by the expression of two chromosomally encoded extracellular ß-lactamases, *bla*_L1_ (a class B metallo-β-lactamase which is not inactivated by aztreonam) and *bla*_L2_ (a class A clavulanic acid susceptible cephalosporinase, which is susceptible to inhibition by serine-β-lactamase inhibitors such as clavulanic acid and avibactam), which would explain the clinical benefit of combined ceftazidime–avibactam if administered in combination with aztreonam [30].

Among its acquired resistance mechanisms, SM can develop cefiderocol resistance through different genetic pathways, such as mutations in the *tonB* gene or in the *smeT* promoter [29]. An increasing presence has also been noted of *sul*1, *sul*2, and *dfrA* genes that have been found both chromosomally and in plasmids, which contribute to SM resistance to trimethoprim-sulfamethoxazole (TMP/SMX). These genes are often associated with Class 1 integrons, which favour their mobility and even carry multiple resistance genes, which are conducive to multi-resistance.

#### 2.2.2. Considerations for Empirical Antimicrobial Decision-Making

This nosocomial pathogen shows low susceptibility to many antibiotics commonly used to empirically treat hospital-acquired infections. In contrast, antimicrobials with a priori in vitro susceptibility, such as TMP/SMX, quinolones, and tetracyclines derivatives (minocycline and tigecycline), are not covered in usual clinical practice in the ICU setting. Gram-negative bacteria are the organisms most commonly responsible for serious infections in the ICU; however, dual empirical coverage usually includes *P. aeruginosa* and *Enterobacteriaceae* as the organisms most frequently involved, whereas SM is responsible for only a minority of cases. SM is, therefore, not typically covered and is likely to be subject to initial inappropriate treatment (ITT). Quinolone is sometimes suggested as an option in empiric combination therapy, such as in the PANNUCI algorithm for nosocomial pneumonia management in the ICU, in which cases SM may be covered [31]. However, quinolones are not always recommended for severe nosocomial infections with sepsis and septic shock, especially given that the resistance rate to ciprofloxacin and levofloxacin in *P. aeruginosa* currently exceeds 30% in most Spanish hospitals [32]. In a large retrospective analysis of ICU patients with bacteraemia and severe sepsis/septic shock caused by a Gram-negative organism, IIT was a key determinant of short-term mortality with a three-fold increase, and MDR was strongly associated with IIT [33]. Notably, SM was specifically identified as a predictor of IIT in this study population. 

SM has been associated with infections after various prolonged antimicrobial treatments, especially with carbapenems, which probably would be selected for SM because of its intrinsic resistance to most of them [9,11]. As well as its prior use perse, cumulative exposure to meropenem has been associated with increased infection risk (hazard ratio, 1.17; 95% CI, 1.01–1.35; *p* = 0.03) in non-critically ill patients, resulting in a 17% increased risk of SM infection with each additional day of meropenem use [14]. Furthermore, a study by Xu et al. concluded that the use of three antibiotics for more than one week was an independent risk factor for SM pneumonia in ICU patients [34]. It seems that not only the type but also the time of exposure and the number of antimicrobials can impact SM infection development. 

Interestingly, SM has been identified as the most frequent carbapenem-resistant (CR) Gram-negative species in hospitalised patients with HAP and VAP [35]. In this retrospective cohort study, which included 8969 patients, a total of 11.8% of isolates were CR, with nearly 60% of this requiring ICU admission. *P. aeruginosa* was the most common Gram-negative pathogen overall (21.1%) but was second to SM (16.8 vs. 38.7%) among CR species. In terms of clinical impact, it is noteworthy that CR was associated with an excess of 3.0 days (95% CI, 1.4–4.6) in overall length of stay and an excess of $8921 (95% CI, 3864–13,977) in hospital costs [35]. 

SM production of inducible carbapenemase, which hydrolyses and detoxifies antibiotics, is also involved in polymicrobial infections, especially among SM and *P. aeruginosa*, although the pathogenic role of SM is difficult to ascertain in this scenario. There is limited understanding of the way antimicrobial resistance evolves in the presence of non-targeted microbial species and how ecological interactions likely alter the effectiveness of antibiotic treatment. CF represents the paradigm of this co-infection, which affects up to a quarter of patients and is often associated with increased mortality [36]. A 24-day-long in vitro experimental study with two-species model CF communities highlighted some aspects related to bacterial eco-evolutionary dynamics in the absence of and under clinically relevant β-lactam imipenem concentrations. Firstly, they found that a clinical SM strain protected sensitive *P. aeruginosa* from imipenem. Secondly, the presence of SM led to an equal or increased rate of imipenem resistance development via parallel loss of function mutations in the OprD porin gene. Finally, and unexpectedly, they observed that resistant *P. aeruginosa* drove SM into extinction due to increased production of pyocyanin in the presence of imipenem, which was cytotoxic to SM [37]. Interestingly, there is some clinical evidence illustrating the way interactions between members can affect the prognosis of this specific polymicrobial infection. SM and *P. aeruginosa* can produce biofilm in the lungs, creating a thriving environment for both. In a Chinese retrospective analysis including severe pneumonia patients, a higher mortality rate and longer hospital stay were reported for those co-infected with *P. aeruginosa* and SM compared with those infected with only one or not infected [38]. 

#### 2.2.3. Related Microbiological Diagnostic Challenges

Caution should be applied when considering in vitro results of SM susceptibility based on antimicrobial susceptibility testing (AST) guidelines (the Clinical and Laboratory Standards Institute [CLSI], the European Committee on Antimicrobial Susceptibility Testing [EUCAST], and Food and Drug Administration [FDA]), which are not standardised. CLSI has established minimum inhibitory concentration (MIC) interpretation criteria for seven antibiotics, including TMP-SMX, ticarcillin–clavulanate, ceftazidime, cefiderocol, levofloxacin, minocycline, and chloramphenicol. In contrast, the EUCAST has defined MIC and disc breakpoints only for TMP-SMX (an antimicrobial with nearly identical breakpoints for EUCAST (>4 mg/L) and CLSI (≥4 mg/L)). The FDA only recognises ceftazidime breakpoints for SM. In real life, only five agents with interpretable antibiotic MIC data are available to clinicians for clinically relevant application in AST (TMP-SMX, ceftazidime, cefiderocol, levofloxacin and minocycline) [29,39]. As a result, differences among SM resistance rates to these antimicrobials become apparent among prevalence studies, and most importantly, the lack of adequate information available may have a considerable impact on patient treatment. 

Accordingly, a recent report including 223 studies in which CLSI recommendations were used evaluated the prevalence of antibiotic resistance in clinical isolates of SM worldwide. The authors observed that the most frequent antibiotic resistance worldwide was against levofloxacin (14.4%) among prevalence studies, with an increased resistance especially in China, and against TMP/SMX (36.84%) among case reports/case series studies, with the highest resistance rate reported in Asia [11]. A more recent worldwide meta-analysis showed a low global prevalence of resistance to TMP-SMX (14.7%), suggesting that this antimicrobial remained effective as a first-line treatment for SM. Other findings were a relatively low resistance rate to levofloxacin (16%), a significant increase in resistance to tigecycline, and the lowest resistance rates documented for minocycline (3%) and cefiderocol (4%) [39]. Regarding the ICU population, the above-mentioned ENVIN-HELICS registry showed a trend towards increased SM resistance to TMP/SMX during the last two years (2022–2023) compared with the pre-pandemic period (2018) (6.2% and 13.5% vs. 0%, respectively), similar to tigecycline (60% and 80% vs. 0%), in contrast to levofloxacin that has remained the same or even decreased (13.65 and 13.3% vs. 15.38%, respectively). Moreover, this report showed an SM resistance of 53.8% to ceftazidime/avibactam and 50% to cefiderocol among 13 and 4 out of 113 tested strains, respectively, within 2022 and 2023 in the participating Spanish ICUs [23]. 

Despite the MDR profile of SM, clinicians should be aware of the epidemiologic and previous colonisation situation in their local institutions to ensure appropriate treatment. Familiarity with microbiological tests, from the simple Gram stain microscopy to new methods for rapid SM identification (such as Matrix-assisted laser desorption ionisation–time of flight [MALDI-TOF] mass spectrometry), as well as a deeper understanding of the value of syndromic panels to detect causative pathogens by molecular methods, are also essential to avoid ITT in the ICU setting (see Table 1). Regarding molecular detection, there are currently few methods that allow the detection of SM directly from biological samples. For the diagnosis of bacterial infections in the lower respiratory tract, the only platform that allows this detection from a direct sample is Unyvero. Furthermore, a recent randomised control trial involving patients with pneumonia in the ICU observed that the growth of ‘off-panel’ pathogens was unusual and most commonly due to *Citrobacter* species and SM. The lack of detection of these organisms by the pneumonia panel did not adversely affect patients’ antimicrobial treatment in this trial [40]. Accordingly, a stewardship strategy for guiding an adjustment of empirical antimicrobial therapy based on the results of rapid microbiological testing like Biofire^®^ Filmarray^®^ Pneumonia Panel plus (BioFire Diagnostics, LLC, Salt Lake City, UT, USA) seems imperative in the management of critically ill patients [41]. Likewise, next-generation metagenomic sequencing (mNGS) has emerged as a promising tool in the field of medicine. Although it shows promising diagnostic performance for severe pneumonia in critically ill patients, facilitating precise diagnoses and treatments, the clinical value of this technology remains controversial due to challenges such as the lack of common standards and guidelines. Continued research and the development of standardized protocols are necessary to maximise its potential and ensure its clinical applicability [42].

### 2.3. Best Evidence-Based Approach to SM Severe Infections. Antimicrobial Stewardship in the Critically Ill

As mentioned above, SM infection management can be hampered either by high-level intrinsic resistance to many antibiotic classes and the increasing occurrence of acquired resistance to the first-line drug cotrimoxazole (TMP/SMX) [3] or by a lack of coverage by the most used empirical antimicrobial strategies for ICU infections, which in turn can cast a shadow over its prognosis.

Different treatment guidelines for infections caused by multidrug-resistant Gram-negative microorganisms have recently been published, including recommendations on SM treatment in guidelines by the Infectious Diseases Society of America (IDSA) and the Society of Infectious Diseases and Clinical Microbiology (SEIMC) [30,50,51]. Published data on the optimal treatment for SM infections are limited. Despite the lack of randomised controlled trials comparing TMP/SMX with any other available treatment for SM infections, it is still currently considered the drug of choice based on high in vitro susceptibility rates [52]. 

For severe infection, the Spanish Society recommends considering combination therapy in immunocompromised patients (strength of recommendation B and quality of evidence II), preferably including TMX/SMX [50]. Likewise, IDSA recommendations from 2022 were recently updated in the 2023 guidelines on the treatment of antimicrobial-resistant Gram-negative infections [53]. The authors directly suggested two approaches: (1) combined use of two of the following agents: TMP-SMX, minocycline/tigecycline, cefiderocol, or levofloxacin, at least until clinical improvement is observed, primarily because of limited and conflicting in vitro and clinical data (limited to observational studies) supporting any individual agent and the need to increase the likelihood of appropriate treatment. (2) The combination of ceftazidime–avibactam plus aztreonam in situations of significant clinical instability following clinical failure with or intolerance to other agents. 

It is noteworthy that the authors recommend a second approach, including ceftazidime–avibactam and aztreonam, in situations of significant clinical instability. This has been considered a reasonable treatment option for severe infections despite limited available clinical data and few case reports, based on the above-mentioned explanation that this combination can overcome the activity of both β-lactamases intrinsic to SM. The IDSA panel expressly advised avoiding ceftazidime as a treatment option because of the presence of β-lactamase genes intrinsic to SM that are expected to render this antimicrobial inactive. The panel also changed the aztreonam regimen in terms of suggested antibiotics dosing from the previous publication [30,53]. Regarding the ICU population, in an Italian case series of 26 COVID-19 ICU patients with VAP due to difficult-to-treat NFGNB, with SM isolated in six of these patients (mostly as part of polymicrobial infection), ceftazidime–avibactam was employed in different regimen combinations. The authors suggest that SM appears as a true pathogen in this patient population, promoting the development of haemorrhagic pneumonia or bacteraemia. A surprising finding was a 30-day overall mortality of 60.8% vs. 16% in an SM-specific subgroup [54]. 

The 2023 American guidelines proposed TMX-SMX and minocycline as the preferred components for combination. Minocycline has slightly more favourable in vitro data, availability of CLSI breakpoints, oral formulation, and likely improved tolerability compared with tigecycline. Moreover, minocycline has been associated with lower adverse effects and mortality than TMP/SMX in a retrospective study dealing with severe patients based on the APACHE-II score [55]. However, tigecycline is also a treatment option for SM infections [30], with extensive penetration into lung tissue at high doses, as demonstrated in critically ill patients [56]. In several series, moreover, the reported MICs for eravacycline are two- to four-fold lower than for tigecycline and frequently mirror minocycline for SM.

Fluoroquinolones are commonly used as an alternative for cases with TMP/SMX-resistant SM or with intolerance due to adverse side effects, especially in HIV-infected patients. Several retrospective observational studies comparing monotherapy with fluoroquinolones and TMP/SMX (with a quarter of patients in the ICU at the time of culture) have suggested that levofloxacin had similar efficacy [57] or even lower OR of death and length of hospital stay than TMP/SMX in patients with lower respiratory tract infections [50,58]. However, because of suboptimal results with fluoroquinolone monotherapy in in vitro studies, known mechanisms of resistance of SM to fluoroquinolones, the emergence of resistance during therapy, and inherent biases in the observational data, the IDSA panel suggests levofloxacin be used only as a component of combination therapy when prescribed for treatment of SM infections [53]. It is noteworthy that treatments (e.g., quinolones) that select mutants leading to multidrug efflux pump overexpression, such as SmeDEF, can reduce SM susceptibility to several antimicrobials simultaneously. This would explain co-resistance to quinolone and cotrimoxazole. However, it does not appear to affect susceptibility to either minocycline or tigecycline, which could thus be candidate antimicrobials in this context [29,52,59]. This could also be the rationale behind the use of antimicrobial combinations in severe infections.

Regarding second-line agents against SM infections, SEIMC experts have recommended their use for their in vitro activity against the isolate (B-II) [50]. In particular, the IDSA proposes cefiderocol because, despite the limited availability of clinical data, in vitro data and animal models are encouraging for its use in treating SM infections, initially in combination with a second active agent [51,53,60]. Clinically, a Spanish retrospective observational study [61] and a French case series [62] assessing the effect of cefiderocol treatment for severe infections due to difficult-to-treat resistant Gram-negative bacilli in critically ill patients reported a 30-day overall mortality rate of nearly 30%. Among the few patients infected with SM (5 and 4, respectively), mortality increased to 80%, although most were immunosuppressed, and related mortality was not specified. Lastly, the ongoing international retrospective medical chart review PROVE study, with a higher sample size regarding SM infections and with more than 70% of patients admitted to ICU, will soon shed further light on the real-life situation.

### 2.4. Preventive Measures: Theoretical Basis for Clinical Formulation of Prevention and Control Strategies to Reduce the Morbidity and Mortality of SM Infection in the ICU

SM is widely distributed in natural environments, such as soil, water, and hospital environments, and can also parasite the human skin, respiratory and digestive tracts. Since environmental and clinical strains are genetically closely related, soil might be a likely source for community-acquired infections [2]. Moreover, sink drains, faucets, water, and sponges have also been identified as environmental sources of SM in hospitals, reflecting the ability of this pathogen to survive on any humid surfaces and colonise them over the long term by forming biofilm and resisting commonly used biocides [2,29,36,63]. SM possesses multiple virulence factors that allow it to colonise and produce infection, and among the most important of these is the production of pili, flagella, fimbria structures, and adhesins, which contribute to adherence, auto-aggregation, and colonisation of biotic and abiotic surfaces. This scenario allows SM to become a potentially important pathogen in the ICU setting, where healthcare-associated infections are a global challenge. 

Guided by whole genome sequencing analysis, there is provided evidence that sink drains serve as antibiotic-resistant organism persistent reservoirs (in particular, SM and *P. aeruginosa* but not *Enterobacterales* species) in ICUs, and moreover that this was associated with human clinical infections, specifically bacteriemia by some *P. aeruginosa* clones [64]. In another previously published study, patients with SM pneumonia were associated with clones originating from sinks and medical devices in a Swedish ICU outbreak [65]. Therefore, disinfection of all possible sources is imperative to avoid healthcare-associated infections with this pathogen [29]. In addition, SM can be transferred via healthcare providers by hand contact. This reinforces the fact that healthcare personnel need to improve infection control practices and keep a close surveillance system [17,66].

Finally, a hospital-acquired BSI has been observed that is seasonal with summer spikes. A national Belgian cohort study on this infection showed associations between ambient climate variables and hospital-acquired BSI incidence rates per microorganism, with Gram-negative but not Gram-positive incidence increasing by 13% for every 5 °C increase in temperature. Stenotrophomonas, Acinetobacter, and Klebsiella spp. demonstrated the highest correlation with increased temperature, and Stenotrophomonas and Bacteroides spp. had higher relative humidity. These factors also warrant further examination to improve patient safety in our units [67].

## 3. Discussion and Future Directions

SM has been identified as a novel nosocomial pathogen associated with life-threatening invasive infections and a high mortality rate in some patient populations, especially those who are severely ill or immunocompromised [10,25]. Clinicians should be made aware of the implications of isolating this organism and should interpret their findings considering clinical settings.

The ICU population is especially vulnerable to this pathogen because of patients’ conditions and an environment conducive to SM colonisation and subsequent patient transmission. As mentioned above, SM can often be recovered from the oropharynx of hospitalised and CF patients. We could hypothesise that critically ill mechanically ventilated patients can mimic CF status, as in both cases, SM causes chronic infection of the airways, which contributes to inflammation and lung damage. Moreover, prolonged ICU stays are accompanied by numerous antimicrobial rounds: these induce ecological effects on microbiota that condition the emergence and frequently the coexistence of MDRB. CF represents the paradigm of bacterial–bacterial community interactions, including Pseudomonas spp. and SM, whose prevalence can be correlated with or sometimes be independent of the use of antimicrobials such as carbapenems [7]. Moreover, poor clinical outcomes in co-infected cases warrant further research to find better antibiotic treatment strategies and case management to reduce co-infection mortality [38].

SM diagnosis also raises many challenges. It is imperative to establish a reproducible, accurate, sensitive, and predictive standard method to detect clinically important susceptibility profiles in contemporary isolates. Moreover, molecular rapid methods should be included in guidelines, but these should preferentially be universal and available for all centres.

As previously mentioned, the use of carbapenem in daily practice in the ICU represents one of the most important risk factors for SM infections from a different perspective. Classically, Del Toro MD et al. hypothesised that antimicrobials would select for SM because of its intrinsic resistance to most antibiotics, thus making colonisation easier to detect with clinical cultures and favouring the possibility of infection in patients debilitated by invasive procedures [9]. Therefore, implementing an antimicrobial stewardship program (ASP) in the ICU attending to a combination of measures related to antimicrobial selection, PK/PD optimisation, and short time duration, is one of the main keys to optimising and dealing with the healthcare problem of increasing antibiotic resistance [68]. Furthermore, ASPs are widely accepted in the ICU, and it has been demonstrated that antimicrobials can be administered without compromising patient safety [69].

It is noteworthy that although TMP-SMX is the treatment of choice for SM infections, it is not routinely included in empirical treatment regimens due both to its adverse event profile and the relative rarity of SM infections in comparison to other Gram-negative bacilli. Consequently, a risk stratification strategy seems important. The use of clinical scoring tools while awaiting final culture results may help identify patients at increased risk for a particular infection, with the intent of reducing time to appropriate antimicrobial therapy. Accordingly, a risk score has been developed that predicts hematologic malignancy patients at increased risk for SM BSI to guide early TMP-SMX therapy [70]. Twenty percent of the 337 patients included were admitted to the ICU. The StenoSCORE, incorporating five variables (AML, absolute neutrophil counts, mucositis, central venous catheter, and carbapenem receipt for ≥3 days), performed moderately well in predicting this infection. Afterward, it was externally validated with a single-centre cohort study with hematologic malignancy, including 36 patients with SM BSI [71]. The authors also evaluated alternative variables that better predicted SM BSI and ICU admission within 12 h of index culture included in the so-called StenoSCORE2. ROC curve analysis of the StenoSCORE2 performance produced an AUC of 0.84 (95% CI 0.76–0.92), which was higher than the previously published StenoSCORE. A StenoSCORE2 ≥ 4 had a sensitivity of 86%, specificity of 76%, and accurately identified 77% of SM BSIs.

Overall, combination therapy with effective antibiotics has been suggested when SM infections are suspected [53]. Unlike the current recommendation of combination therapy for empiric treatment of suspected or proven septic shock in ICU patients and monotherapy for definitive treatment when culture results and susceptibility tests are available, even for NFGNB, combination therapy could be discussed in patients with proven difficult-to-treat pathogens such as SM or carbapenem-resistant *Enterobacteriaceae* during ASP in the ICU, following clinical practice guidelines [68,72].

Opening further lines of research may help control the spread of SM in the ICU setting, with the optimisation of infection control policies deserving special attention [17].

Table 2 shows the most relevant findings and evidence-based key messages regarding severe SM infections in the critically ill population.

This paper has several limitations inherent to narrative review methodology, such as potential incomplete coverage, lack of quantitative analysis, and positive results bias. Heterogeneity in study designs and the lack of explicit criteria for study selection may have also introduced some bias. Nevertheless, to our knowledge, no previous study has comprehensively evaluated evidence on Stenotrophomonas maltophilia infections in critical patients. However, data are limited, and relevant clinical questions remain outstanding, such as the microbiological diagnostic challenges, proper antimicrobial stewardship in the critically ill, and concrete preventive measures in these settings.

## 4. Conclusions

SM infection remains a challenge for critical care physicians. A high index of suspicion and deep knowledge of both the local epidemiological situation and the new rapid diagnostic tools linked to the implementation of an ICU-ASP in daily practice, as well as control measures, would improve the ominous prognosis of this bacterium’s presence in the ICU setting.

## Figures and Tables

**Table 1:** New microbiological diagnostic methods for rapid SM identification.

Diagnostic Test	Pathogen/ Resistance	Sample Type	Time Required to Detection (Hours)	Detection Limit	Sensitivity (%)	Specificity (%)	Robustness	Reference
Unyvero A50 System HPN (Curetis, Holzgerlingen, Germany)	21 Gram-negative and Gram-positive bacteria/17 antibiotic-resistant markers	Sputum, bronchoalveolar lavage, tracheal and bronchial secretions	<5 h	NR	84	98	NR	Sun et al., 2021 [43]
Unyvero A50 System BCU (Curetis)	34 Pathogens, 16 antibiotic resistance markers	Positive blood culture	<5 h	NR	96.8	99.8	NR	Burrack-Lange et al., 2018 [44]
Biofire®Filmarray® BCID2 (bioMeriéux, Craponne, France)	33 Gram-negative, Gram-positive bacteria, Candida spp., and 10 antibiotic-resistant markers	Positive blood culture	1 h	NR	>80 for SM	99.8	NR	Peri et al., 2022 [45]
Sepsis Flow Chip (VITRO Master Diagnóstica, Granada, Spain)	17 Gram-negative and Gram-positive bacteria, 1 yeast, and 19 antibiotic-resistant markers	Positive blood culture	3 h	NR	100 for SM	100	NR	Galiana et al., 2017 [46]
hemoFISH (Miacom Diagnostics, Düsseldorf, Germany)	19 Gram-negative and Gram-positive bacteria	Positive blood culture	>5 h	NR	<80–90 for SM	>95	NR	Reitz et al., 2018 [47]
Magicplex Sepsis Real-Time (Seegene, Seoul, Republic of Korea)	73 Gram-positive, 12 Gram-negative, 6 fungal, and 3 antibiotic-resistant markers	Whole blood	3–6 h	NR	65	92	NR	Carrara et al., 2013 [48]
MALDI Biotyper System (Bruker, Billerica, MA, USA)	Bacterial and fungal species or species groups	A single colony from isolate positive culture or positive whole blood cultures	minutes	-	73 for SM	99	NR	Gautam et al., 2017 [49]
LiDia-SEQ NGS (DNA Electronics Ltd., Carlsbad, CA, USA)	Bacterial and fungal pathogens	Whole blood	>4 h	1 CFU/mL	NR	NR	NR	Not found

Note: SM: *Stenothophomonas maltophilia*; NR: not reported; CFU: colony forming unit.

**Table 2:** Most relevant findings and key messages regarding severe SM infections in the critically ill population.

Scenario	Clinical Evidence
Epidemiology	Recent surveillance data indicate that SM accounts for less than 3% of all strains of healthcare-associated infections, a percentage that doubles in the case of VAP [5,22,23]. Crude 28-day mortality is 54.8% [13], increasing to 90% if hemorrhagic pneumonia is present [25], and it is further increased with co-infection by *Pseudomonas aeruginosa* [38]
Risk factors	Importance of stratification strategy: pre-existing medical conditions (COPD, malignancy); length of ICU stay; invasive procedures and antimicrobial agents, especially CP [15]. StenoSCORE2 for SM bacteriemia prediction [71]
Colonisation	Oral SM relative abundance of 36% predicts infection [14]. Previous SM colonisation was found in 80% of patients with pneumonia and 67% of patients with bacteremia caused by SM [6,16]
Antibiotic resistance	SM is intrinsically resistant to a wide range of commonly used antibiotics [29]. The resistance surveillance data reflect an increase, especially for cotrimoxazole and tigecycline [11,23,39]
Diagnostic challenges	There is no consensus among antimicrobial susceptibility testing guidelines [29,39]. An ICU-ASP for guiding an adjustment of empirical antimicrobial therapy based on rapid microbiological testing seems imperative [41,68]
Empirical therapy	SM predicts initially inappropriate antibiotic therapy [33]
Directed therapy	Combination therapy is recommended until clinical improvement [51,53]
Prevention measures	SM establishes ambiental ICU reservoirs and is related to patient infection [64,65]. Optimisation of infection control policies deserves special attention [17]

Note: SM: *Stenothophomonas maltophilia*; VAP: ventilator-associated pneumonia; COPD: chronic obstructive pulmonary disease; ICU: intensive care unit; CP: carbapenems; ASP: antimicrobial stewardship program.

## References

[1] Ryan R., Monchy S., Cardinale M., Taghavi S., Crossman L., Avison M., Berg G., van der Lelie D., Dow J.M. (2009). The versatility and adaptation of bacteria from the genus Stenotrophomonas. Nat. Rev. Microbiol..

[2] Brooke J.S. (2012). Stenotrophomonas maltophilia: An emerging global opportunistic pathogen. Clin. Microbiol. Rev..

[3] Looney W.J., Narita M., Mühlemann K. (2009). *Stenotrophomonas maltophilia*: An emerging opportunist human pathogen. Lancet Infect. Dis..

[4] Fihman V., Le Monnier A., Corvec S., Jaureguy F., Tankovic J., Jacquier H., Carbonnelle E., Bille E., Illiaquer M., Cattoir V. (2012). *Stenotrophomonas maltophilia*—The most worrisome threat among unusual non-fermentative gram-negative bacilli from hospitalized patients: A prospective multicenter study. J. Infect..

[5] Fupin H., Yan G., Demei Z., Fu W., Xiaofei J. (2021). CHINET surveillance of bacterial resistance: Results of 2020. Chin J Infect Chemother..

[6] Nseir S., Pompeo C., Brisson H., Dewavrin F., Tissier S., Diarra M., Boulo M., Durocher A. (2006). Intensive care unit-acquired Stenotrophomonas maltophilia: Incidence, risk factors, and outcome. Crit. Care..

[7] Koulenti D., Vandana K.E., Rello J. (2023). Current viewpoint on the epidemiology of nonfermenting Gram-negative bacterial strains. Curr. Opin. Infect. Dis..

[8] Horan T.C., Andrus M., Dudeck M.A. (2008). CDC/NHSN surveillance definition of health care-associated infection and criteria for specific types of infections in the acute care setting. Am. J. Infect. Control.

[9] Del Toro M.D., Rodríguez-Bano J., Herrero M., Rivero A., García-Ordoñez M.A., Corzo J., Pérez-Cano R., Grupo Andaluz para el Estudio de las Enfermedades Infecciosas (2002). Clinical epidemiology of Stenotrophomonas maltophilia colonization and infection: A multicenter study. Medicine.

[10] Falagas M., Kastoris A., Vouloumanou E., Rafailidis P., Kapaskelis A., Dimopoulos G. (2009). Attributable mortality of Stenotrophomonas maltophilia infections: A systematic review of the literature. Future Microbiol..

[11] Dadashi M., Hajikhani B., Nazarinejad N., Noorisepehr N., Yazdani S., Hashemi A., Hashemizadeh Z., Goudarzi M., Fatemeh S. (2023). Global prevalence and distribution of antibiotic resistance among clinical isolates of Stenotrophomonas maltophilia: A systematic review and meta-analysis. J. Glob. Antimicrob. Resist..

[12] Laing F.P.Y., Ramotar K., Read R.R., Alfieri N., Kureishi A., Henderson E.A., Louie T.J. (1995). Molecular epidemiology of Xanthomonas maltophilia colonization and infection in the hospital environment. J. Clin. Microbiol..

[13] Dimopoulos G., Garnacho-Montero J., Paramythiotou E., Gutierrez-Pizarraya A., Gogos C., Adriansen-Pérez M., Diakaki C., Matthaiou D.K., Poulakou G., Akinosoglou K. (2023). Upraising Stenotrophomonas maltophilia in Critically Ill Patients: A New Enemy?. Diagnostics.

[14] Aitken S.L., Sahasrabhojane P.V., Kontoyiannis D.P., Savidge T.C., Arias C.A., Ajami N.J., Shelburne S.A., Galloway-Peña J.R. (2021). Alterations of the Oral Microbiome and Cumulative Carbapenem Exposure Are Associated With Stenotrophomonas maltophilia Infection in Patients With Acute Myeloid Leukemia Receiving Chemotherapy. Clin. Infect. Dis..

[15] Wang N., Tang C., Wang L. (2022). Risk Factors for Acquired Stenotrophomonas maltophilia Pneumonia in Intensive Care Unit: A Systematic Review and Meta-Analysis. Front Med..

[16] Hotta G., Matsumura Y., Kato K., Nakano S., Yunoki T., Yamamoto M., Nagao M., Ito Y., Takakura S., Ichiyama S. (2014). Risk factors and outcomes of Stenotrophomonas maltophilia bacteraemia: A comparison with bacteraemia caused by Pseudomonas aeruginosa and Acinetobacter species. PLoS ONE..

[17] Álvarez-Lerma F., Catalán-González M., Álvarez J., Sánchez-García M., Palomar-Martínez M., Fernández-Moreno I., Garnacho-Montero J., Barcenilla-Gaite F., García R., Aranaz-Andrés J. (2023). Impact of the "Zero Resistance" program on acquisition of multidrug-resistant bacteria in patients admitted to Intensive Care Units in Spain. A prospective, intervention, multimodal, multicenter study. Med. Intensiv..

[18] Detsis M., Karanika S., Mylonakis E. (2017). ICU Acquisition Rate, Risk Factors, and Clinical Significance of Digestive Tract Colonization With Extended-Spectrum Beta-Lactamase-Producing Enterobacteriaceae: A Systematic Review and Meta-Analysis. Crit. Care Med..

[19] Torres I., Huntley D., Tormo M., Calabuig M., Hernández-Boluda J.C., Terol M.J., Carretero C., de Michelena P., Pérez A., Piñana J.L. (2022). Multi-body-site colonization screening cultures for predicting multi-drug resistant Gram-negative and Gram-positive bacteremia in hematological patients. BMC Infect. Dis..

[20] Scheich S., Koenig R., Wilke A.C., Lindner S., Reinheimer C., Wichelhaus T.A., Hogardt M., Kempf V.A.J., Kessel J., Weber S. (2018). Stenotrophomonas maltophilia colonization during allogeneic hematopoietic stem cell transplantation is associated with impaired survival. PLoS ONE..

[21] Ishikawa K., Nakamura T., Kawai F., Uehara Y., Mori N. (2022). Stenotrophomonas maltophilia Infection Associated with COVID-19: A Case Series and Literature Review. Am. J. Case Rep..

[22] European Centre for Disease Prevention and Control (2024). Point Prevalence Survey of Healthcare-Associated Infections and Antimicrobial Use in European Acute Care Hospitals.

[23] ENVIN-HELICS. http://hws.vhebron.net/envin-helics/.

[24] Guo L., Li H., Li Q., Juan J. (2014). Antimicrobial drug-sensitivity and clinical risk factors of stenotro-phomnas maltophilia in the neurological intensive care unit. J. Xiangnan Univ. (Med. Sci.).

[25] Huang C., Kuo S., Lin L. (2024). Hemorrhagic Pneumonia Caused by Stenotrophomonas maltophilia in Patients with Hematologic Malignancies-A Systematic Review and Meta-Analysis. Medicina.

[26] Gutierrez C., Pravinkumar E., Balachandran D., Schneider V. (2016). Fatal hemorrhagic pneumonia: Don’t forget Stenotrophomonas maltophilia. Respir. Med. Case Rep..

[27] Imoto W., Yamada K., Yamairi K., Shibata W., Namikawa H., Yukawa S., Yoshii N., Nakaie K., Hirose A., Koh H. (2020). Clinical Characteristics of Rapidly Progressive Fatal Hemorrhagic Pneumonia Caused by Stenotrophomonas maltophilia. Intern. Med..

[28] Windhorst S., Frank E., Georgieva D.N., Genov N., Buck F., Borowski P., Weber W. (2022). The major extracellular protease of the nosocomial pathogen Stenotrophomonas maltophilia: Characterization of the protein and molecular cloning of the gene. J. Biol. Chem..

[29] Mojica M.F., Humphries R., Lipuma J.J., Mathers A.J., Rao G.G., Shelburne S.A., Fouts D.E., Van Duin D., Bonomo R.A. (2022). Clinical challenges treating Stenotrophomonas maltophilia infections: An update. JAC Antimicrob. Resist..

[30] Tamma P.D., Aitken S.L., Bonomo R.A., Mathers A.J., van Duin D., Clancy C.J. (2022). Infectious Diseases Society of America Guidance on the Treatment of AmpC beta-Lactamase-Producing Enterobacterales, Carbapenem-Resistant Acinetobacter baumannii, and Stenotrophomonas maltophilia Infections. Clin. Infect. Dis..

[31] Zaragoza R., Vidal-Cortés P., Aguilar G., Borges M., Diaz E., Ferrer R., Maseda E., Nieto M., Nuvials F.X., Ramirez P. (2020). Update of the treatment of nosocomial pneumonia in the ICU. Crit. Care..

[32] Mensa J., Barberán J., Ferrer R., Borges M., Rascado P., Maseda E., Oliver A., Marco F., Adalia R., Aguilar G. (2021). Recommendations for antibiotic selection for severe nosocomial infections. Rev. Esp. Quimioter..

[33] Zilberberg M.D., Shorr A.F., Micek S.T., Vazquez-Guillamet C., Kollef M.H. (2014). Multi-drug resistance, inappropriate initial antibiotic therapy and mortality in Gram-negative severe sepsis and septic shock: A retrospective cohort study. Crit. Care..

[34] Xu N.L., Shi S.J., Lai Z.S., Li H.R., Lian S.Q., Chen Y.S. (2011). A case-control study on the risk factors for lower respiratory tract infection by Stenotrophomonas maltophilia in a medical intensive care unit. Chin. J. Tuberc. Respir. Dis..

[35] Zilberberg M.D., Nathanson B.H., Sulham K., Fan W., Shorr A.F. (2019). A novel algorithm to analyze epidemiology and outcomes of carbapenem resistance among patients with hospital-acquired and ventilator-associated pneumonia: A retrospective cohort study. Chest.

[36] Sherrard L.J., Tunney M.M., Elborn J.S. (2014). Antimicrobial resistance in the respiratory microbiota of people with cystic fibrosis. Lancet.

[37] Quinn A.M., Bottery M.J., Thompson H., Friman V.P. (2022). Resistance evolution can disrupt antibiotic exposure protection through competitive exclusion of the protective species. ISME J..

[38] Yin C., Yang W., Meng J., Lv Y., Wang J., Huang B. (2017). Co-infection of Pseudomonas aeruginosa and Stenotrophomonas maltophilia in hospitalised pneumonia patients has a synergic and significant impact on clinical outcomes. Eur. J. Clin. Microbiol. Infect. Dis..

[39] Bostanghadiri N., Sholeh M., Navidifar T., Dadgar-Zankbar L., Elahi Z., van Belkum A., Darban-Sarokhalil D. (2024). Global mapping of antibiotic resistance rates among clinical isolates of Stenotrophomonas maltophilia: A systematic review and meta-analysis. Ann. Clin. Microbiol. Antimicrob..

[40] Poole S., Tanner A.R., Naidu V.V., Borca F., Phan H., Saeed K., Grocott M.P.W., Dushianthan A., Moyses H., Clark T.W. (2022). Molecular point-of-care testing for lower respiratory tract pathogens improves safe antibiotic de-escalation in patients with pneumonia in the ICU: Results of a randomised controlled trial. J. Infect..

[41] Clari M.A., Carbonell N., Albert E., Navarro D. (2023). Proposal for antimicrobial therapy stewardship of lower respiratory tract infection in mechanically-ventilated patients based upon the Biofire® Filmarray® Pneumonia Plus panel results. Enfermedades Infecc. Microbiol. Clin..

[42] Lv M., Zhu C., Zhu C., Yao J., Xie L., Zhang C., Huang J., Du X., Feng G. (2023). Clinical values of metagenomic next-generation sequencing in patients with severe pneumonia: A systematic review and meta-analysis. Front. Cell Infect. Microbiol..

[43] Sun L., Li L., Du S., Liu Y., Cao B. (2021). An evaluation of the Unyvero pneumonia system for rapid detection of microorganisms and resistance markers of lower respiratory infections-a multicenter prospective study on ICU patients. Eur. J. Clin. Microb. Infect. Dis..

[44] Burrack-Lange S.C., Personne Y., Huber M., Winkler E., Weile J., Knabbe C., Görig J., Rohde H. (2018). Multicenter assessment of the rapid Unyvero Blood Culture molecular assay. J. Med. Microbiol..

[45] Peri A.M., Ling W., Furuya-Kanamori L., Harris P.N.A., Paterson D.L. (2022). Performance of BioFire Blood Culture Identification 2 Panel (BCID2) for the detection of bloodstream pathogens and their associated resistance markers: A systematic review and meta-analysis of diagnostic test accuracy studies. BMC Infect. Dis..

[46] Galiana A., Coy J., Gimeno A., Guzman N.M., Rosales F., Merino E., Royo G., Rodríguez J.C. (2017). Evaluation of the Sepsis Flow Chip assay for the diagnosis of blood infections. PLoS ONE..

[47] Reitz A., Poppert S., Rieker M., Frickmann H. (2018). Evaluation of FISH for Blood Cultures under Diagnostic Real-Life Conditions. Eur. J. Microbiol. Immunol..

[48] Carrara L., Navarro F., Turbau M., Seres M., Morán I., Quintana I., Martino R., González Y., Brell A., Cordon O. (2013). Molecular diagnosis of bloodstream infections with a new dual-priming oligonucleotide-based multiplex PCR assay. J. Med. Microbiol..

[49] Gautam V., Sharma M., Singhal L., Kumar S., Kaur P., Tiwari R., Ray P. (2017). MALDI-TOF mass spectrometry: An emerging tool for unequivocal identification of non-fermenting Gram-negative bacilli. Indian. J. Med. Res..

[50] Pintado V., Ruiz-Garbajosa P., Aguilera-Alonso D., Baquero-Artigao F., Bou G., Cantón R., Grau S., Gutiérrez-Gutiérrez B., Larrosa N., Machuca I. (2023). Executive summary of the consensus document of the Spanish Society of Infectious Diseases and Clinical Microbiology (SEIMC) on the diagnosis and antimicrobial treatment of infections due to carbapenem-resistant Gram-negative bacteria. Enfermedades Infecc. Microbiol. Clin..

[51] Cantón R., Ruiz-Garbajosa P. (2023). Treatment guidelines for multidrug-resistant Gram-negative microorganisms. Rev. Esp. Quimioter..

[52] Wei C., Ni W., Cai X., Zhao J., Cui J. (2016). Evaluation of Trimethoprim/Sulfamethoxazole (SXT), Minocycline, Tigecycline, Moxifloxacin, and Ceftazidime Alone and in Combinations for SXT-Susceptible and SXT-Resistant Stenotrophomonas maltophilia by In Vitro Time-Kill Experiments. PLoS ONE..

[53] Tamma P.D., Aitken S.L., Bonomo R.A., Mathers A.J., van Duin D., Clancy C.J. (2023). Infectious Diseases Society of America 2023 Guidance on the Treatment of Antimicrobial Resistant Gram-Negative Infections. Clin. Infect. Dis..

[54] Burastero G.J., Orlando G., Santoro A., Menozzi M., Franceschini E., Bedini A., Cervo A., Faltoni M., Bacca E., Biagioni E. (2022). Ceftazidime/Avibactam in Ventilator-Associated Pneumonia Due to Difficult-to-Treat Non-Fermenter Gram-Negative Bacteria in COVID-19 Patients: A Case Series and Review of the Literature. Antibiotics.

[55] Junco S.J., Bowman M.C., Turner R.B. (2021). Clinical outcomes of Stenotrophomonas maltophilia infection treated with trimethoprim/sulfamethoxazole, minocycline, or fluoroquinolone monotherapy. Int. J. Antimicrob. Agents..

[56] De Pascale G., Lisi L., Pia Ciotti G.M., Vallecoccia M.S., Cutuli S.L., Cascarano L., Gelormini C., Bello G., Montini L., Carelli S. (2020). Pharmacokinetics of high-dose tigecycline in critically ill patients with severe infections. Ann. Intensive Care..

[57] Wang Y.L., Scipione M.R., Dubrovskaya Y., Papadopoulos J. (2014). Monotherapy with fluoroquinolone or trimethoprim-sulfamethoxazole for treatment of Stenotrophomonas maltophilia infections. Antimicrob. Agents Chemother..

[58] Sarzynski S.H., Warner S., Sun J., Matsouaka R., Dekker J.P., Babiker A., Li W., Lai Y.L., Danner R.L., Fowler V.G. (2022). Trimethoprim-Sulfamethoxazole Versus Levofloxacin for Stenotrophomonas maltophilia Infections: A Retrospective Comparative Effectiveness Study of Electronic Health Records from 154 US Hospitals. Open Forum Infect. Dis..

[59] Sánchez M.B., Martínez J.L. (2015). The efflux pump SmeDEF contributes to trimethoprim-sulfamethoxazole resistance in Stenotrophomonas maltophilia. Antimicrob. Agents Chemother..

[60] Gijón D., García-Castillo J., Fernández-López M.C., Bou G., Siller M., Calvo-Montes J., Pitart C., Vila J., Torno N., Gimeno C. (2024). In vitro activity of cefiderocol and other newly approved antimicrobials against multi-drug resistant Gram-negative pathogens recovered in intensive care units in Spain and Portugal. Rev. Esp. Quimioter..

[61] Balandín B., Pintado V., Pérez-Pedrero M.J., Martínez-Sagasti F., Sancho-González M., Soriano-Cuesta C., Gesso C.M., Chicot M., de Luna R.R., Asensio-Martín M.J. (2024). Multicentre study of cefiderocol for treatment of Gram-negative bacteria infections in critically ill patients. Int. J. Antimicrob. Agents..

[62] Wicky P.H., Poiraud J., Alves M., Patrier J., d’Humières C., Lê M., Kramer L., de Montmollin É., Massias L., Armand-Lefèvre L. (2023). Cefiderocol Treatment for Severe Infections due to Difficult-to-Treat-Resistant Non-Fermentative Gram-Negative Bacilli in ICU Patients: A Case Series and Narrative Literature Review. Antibiotics.

[63] Guyot A., Turton J.F., Garner D. (2013). Outbreak of Stenotrophomonas maltophilia on an Intensive Care Unit. J. Hosp. Infect..

[64] Sukhum K.V., Newcomer E.P., Cass C., Wallace M.A., Johnson C., Fine J., Sax S., Barlet M.H., Burnham C.D., Dantas G. (2022). Antibiotic-resistant organisms establish reservoirs in new hospital built environments and are related to patient blood infection isolates. Commun Med..

[65] Gideskog M., Welander J., Melhus Å. (2020). Cluster of S. maltophilia among patients with respiratory tract infections at an intensive care unit. Infect. Prev. Pract..

[66] Baidya A., Kodan P., Fazal F., Tsering S., Menon P.R., Jorwal P., Chowdhury U.K. (2019). Stenotrophomonas maltophilia: More than just a colonizer! Indian. J. Crit. Care Med..

[67] Blot K., Hammami N., Blot S., Vogelaers D., Lambert M.L. (2022). Seasonal variation of hospital-acquired bloodstream infections: A national cohort study. Infect. Control Hosp. Epidemiol..

[68] Mokrani D., Chommeloux J., Pineton de Chambrun M., Hékimian G., Luyt C.E. (2023). Antibiotic stewardship in the ICU: Time to shift into overdrive. Ann. Intensive Care..

[69] Pérez-Torres D., Tamayo-Lomas L.M., Domínguez-Gil González M., Almendros-Muñoz R., Sacristán-Salgado M.A., González-González E., Berezo-García J.A., Díaz-Rodríguez C., Canas-Pérez I., Lorenzo-Vidal B. (2023). Antimicrobial stewardship program in an Intensive Care Unit: A retrospective observational analysis of the results 15 months after its implementation. Rev. Esp. Quimioter..

[70] Karaba S.M., Goodman K.E., Amoah J., Cosgrove S.E., Tamma P.D. (2021). StenoSCORE: Predicting Stenotrophomonas maltophilia Bloodstream Infections in the Hematologic Malignancy Population. Antimicrob. Agents Chemother..

[71] Gill E.L., Gill C.M., McEvoy C. (2024). Validation of a Stenotrophomonas maltophilia bloodstream infection prediction score in the hematologic malignancy population. Ann. Hematol..

[72] Torres A., Niederman M.S., Chastre J., Ewig S., Fernandez-Vandellos P., Hanberger H., Kollef M., Li Bassi G., Luna C.M., Martin-Loeches I. (2017). International ERS/ESICM/ESCMID/ALAT guidelines for the management of hospital-acquired pneumonia and ventilator associated pneumonia: Guidelines for the management of hospital acquired pneumonia (HAP)/ventilator-associated pneumonia (VAP) of the European Respiratory Society (ERS), European Society of Intensive Care Medicine (ESICM), European Society of Clinical Microbiology and Infectious Diseases (ESCMID) and Asociacion Latinoamericana del Torax (ALAT). Eur. Respir. J..

